# Efficient VANET safety message delivery and authenticity with privacy preservation

**DOI:** 10.7717/peerj-cs.519

**Published:** 2021-05-04

**Authors:** Taha M. Mohamed, Islam Z. Ahmed, Rowayda A. Sadek

**Affiliations:** 1 Faculty of Computers and Artificial Intelligence, Helwan University, Cairo, Egypt; 2 Faculty of Business, University of Jeddah, Jeddah, Kingdom of Saudi Arabia (KSA)

**Keywords:** VANETs, Authentication, Privacy, Security, Diffie–Hellman, RSA, CRT-RSA, RSSI

## Abstract

Vehicular ad-hoc networks (VANETs) play an essential role in the development of the intelligent transportation system (ITS). VANET supports many types of applications that have strict time constraints. The communication and computational overheads are minimal for these computations and there are many security requirements that should be maintained. We propose an efficient message authentication system with a privacy preservation protocol. This protocol reduces the overall communication and computational overheads. The proposed protocol consists of three main phases: the group registration phase, send/receive messages phase, and the leave/join phase. For cryptography algorithms, we combined symmetric and asymmetric key algorithms. The symmetric key was generated and exchanged without using the Diffie–Hellman (DH) protocol. Furthermore, we used an efficient version of the RSA algorithm called CRT-RSA. The experimental results showed that the computational overhead in the registration phase was significantly reduced by 91.7%. The computational overhead for sending and receiving the non-safety message phase was reduced by 41.2% compared to other existed protocols. Moreover, our results showed that the time required to broadcast a safety and non-safety group message was below 100 ms and 150 ms, respectively. The average computational time of sending and receiving a one-to-one message was also calculated. The proposed protocol was also evaluated with respect to performance and security and was shown to be invulnerable to many security attacks.

## Introduction

Vehicular ad hoc networks (VANETs) provide wireless communication between moving nodes (vehicles) and an installed infrastructure. VANET has the primary role in intelligent transportation systems (ITS). These systems provide traffic management services that aim to reduce road accidents and reduce traffic congestion (Shanmugasundaram et al., 2017). VANET is a subclass of mobile ad hoc network (MANET). There are many similarities between them: a short communication range, low bandwidth, self-organization, and self-management (Shanmugasundaram et al., 2017). However, VANET has unique characteristics that differ from MANET. These characteristics include: a highly dynamic topology, large network scale, and frequently disconnected connections (Islam, Taha & Rowayda, 2017). Unlike wireless sensor networks (WSNs), VANETs have sufficient energy needed for computations and communications; energy conservation is not an important factor for VANETs (Islam, Taha & Rowayda, 2017).

**Figure 1:**
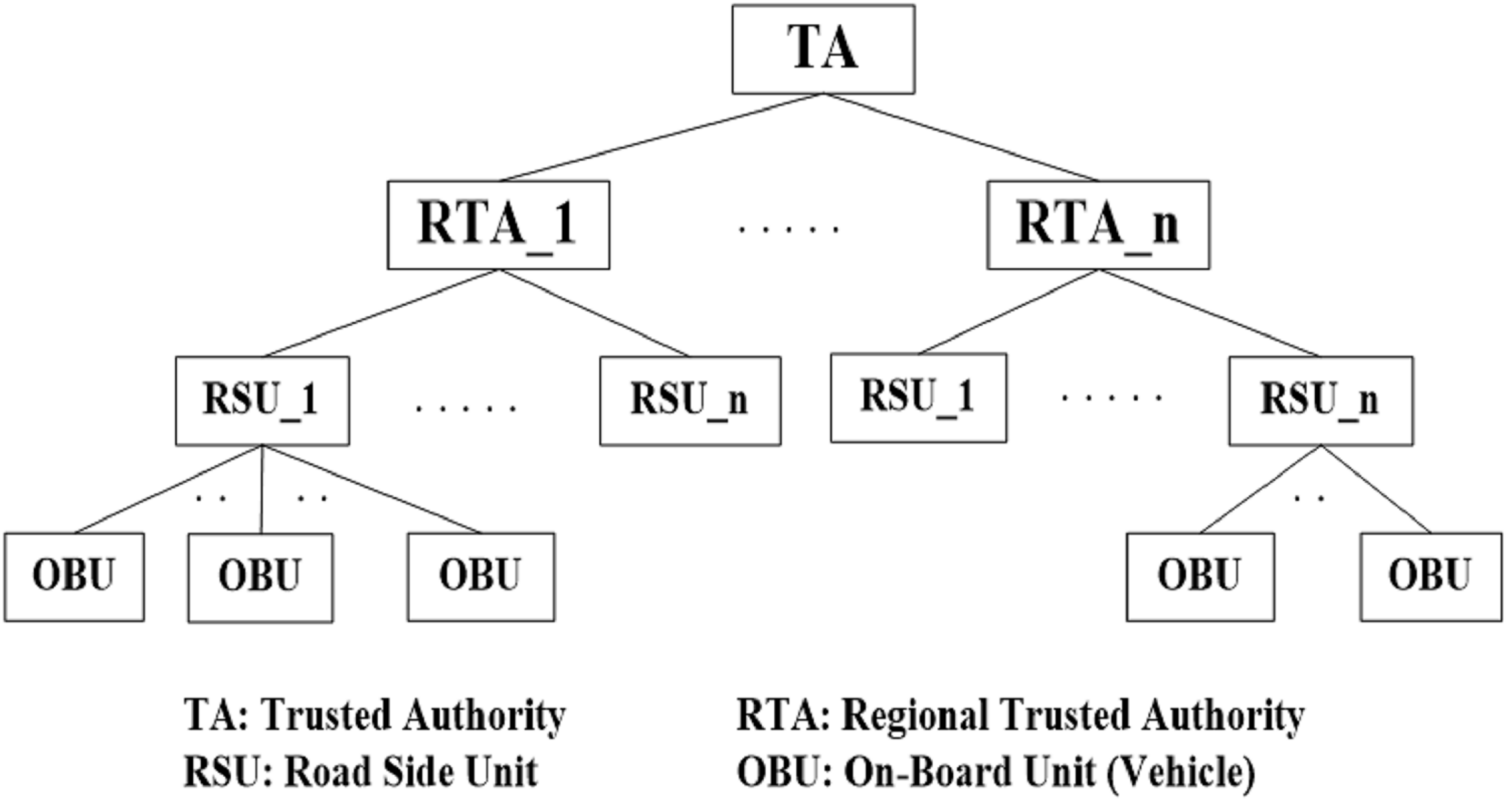
VANET architecture.

VANET has three main components: the Trusted Authority (TA), Roadside Unit (RSU), and On-Board Unit (OBU) as shown in Fig. 1. The Trusted Authority is the first component that acts as a third party. TA issues and revokes vehicle certificates and provides RSUs and OBUs with security parameters such as key pairs (public/private) (Lim & Manivannan, 2016). Furthermore, it maintains an identity list for all vehicles and the certificate revocation list (CRL) of illegal (malicious) vehicles. In VANETs, each region has a regional trusted authority (RTA) that controls a specific area (Jiayu & Tianhan, 2020). RTAs are used to alleviate communication and computational overhead on the TA. Moreover, each RTA manages and controls all RSUs allocated in its region. RTAs also provide RSUs with the recent certificate revocation list of all malicious vehicles (Jiayu & Tianhan, 2020) and when a vehicle receives false information, this information will be reported to the RSU to be actioned. The RSU will then report the malicious vehicle certificate to the RTA (Jiayu & Tianhan, 2020).

The second component is the OBU mounted on each vehicle. The OBU is a wave device used for exchanging information with other OBUs. OBUs can exchange information with roadside units (RSUs). This process uses a dedicated short-range communication (DSRC) (El-Rewini et al., 2020).

The last component is the RSU. The TA usually installs RSUs in a specific location on the road, such as parking spaces or road junctions. RSUs are used to provide safety and entertainment services to legal vehicles on the road. All RSUs can communicate directly with each other using wired or wireless communications. Additionally, they can communicate indirectly through the RTA (El-Rewini et al., 2020).

This distributed architecture (TA-RTA-RSU) has the advantage of low communication and computational overheads (Jiang, Ge & Shen, 2020). This architecture is analogous to the Edge-Fog-Cloud computing architecture that solves many issues related to IoT services. In the proposed architecture, RSU, RTA, and TA nodes are like Edge, Fog, and Cloud nodes, respectively. This distributed architecture additionally improves the overall end-to-end delay and security by shifting data processing to units closer to nodes.

**Figure 2:**
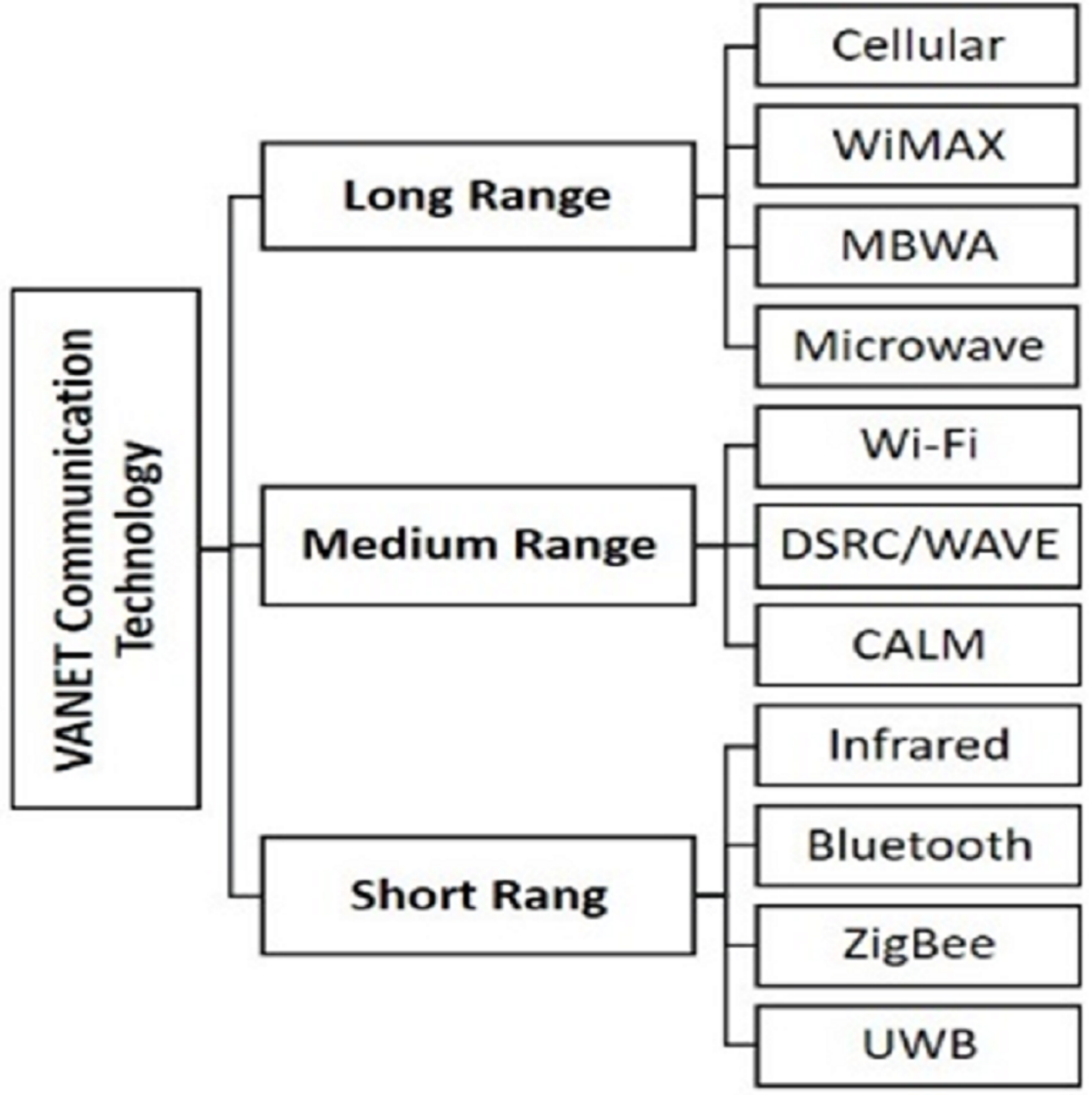
VANET communication technologies.

In VANET, vehicles can use various wireless technologies to communicate with each other and communicate with RSUs (Ali, Hassan & Li, 2019). These communication technologies can be classified into three types based on the signal range shown in Fig. 2. DSRC is also known as 802.11p, which modifies the IEEE 802.11 on MAC and PHY layers (Al-Sultan et al., 2014; Ismath et al., 2019). Wireless Access for Vehicular Environments (WAVE) is also a version of the IEEE 802.11 standard. It is required to assist the short-range communications in ITS applications. VANETs use the DSRC/WAVE, which is the basic communication technology in VANET (Bitam & Mellouk, 2014).

VANETs support a large number of applications. These applications can be classified into two main types (Al-Sultan et al., 2014; Al-ani et al., 2020). The first type is the comfort/entertainment applications that aim to provide drivers with useful road information. This information may include the location of gas stations, hotels, parking, restaurants, Internet access, and road traffic status (Al-Sultan et al., 2014; Malhi, Batra & Pannu, 2020). The second type is the safety applications that aim to enhance road safety. Safety applications include: intersection collision avoidance, traffic sign violation warning, accident notification, and U-turn assistance (Al-ani et al., 2020; Goyal, Agarwal & Tripathi, 2019). Safety applications require hard real-time constraints and a safety message must be sent and received within 100 ms (Hu & Laberteaux, 2006).

RSUs have specific roadside locations with separation distances of two to three km between each two RSUs (Chaurasia & Verma, 2011). The average traveling speed of each vehicle is assumed to be 20 m/s. This means the vehicle will pass between any two RSUs in 4 to 5 min (Hu & Laberteaux, 2006). Hence, the message should be delivered and verified in a few milliseconds.

There are a number of security issues to be addressed in VANETs, including message authenticity with privacy preservation, confidentiality, integrity, and non-repudiation (Manvi & Tangade, 2017). We determined an efficient protocol for message delivery and authentication while maintaining privacy. Our proposed protocol is resistant to many attacks and satisfies all security requirements by reducing the communication and computational overhead.

Our findings are organized as follows: in “Related Work” we review the previous work; in “System Assumptions and Objectives” we introduce the system’s assumptions and objectives; in “The Proposed Protocol” we detail the proposed protocol; in “Simulation Results and Discussion” we show, analyze, and discuss the experimental results; in “Security Analysis” we analyze the security requirements and attack resistance of the proposed protocol; in “Conclusion” we provide the discussion and conclusions.

## Related work

This section outlines the previous works related to VANET message authentication with privacy preservation. A number of authentication schemes have been proposed in the literature. The existing authentication schemes can be classified as: symmetric key, asymmetric key, and hybrid key authentication schemes.

### Symmetric key authentication schemes

Hu & Laberteaux (2006) proposed a Timed Efficient Synchronous Loss-Tolerant Authentication protocol (TESLA) that first broadcasts its MAC's encrypted message, then, after a specified period, the sending vehicle broadcasts the encryption key to all receiving vehicles. This process will help verify message authenticity and then decrypt the message. However, this scheme is not suitable for safety applications that have strict time constraints. Manvi & Tangade (2017) proposed a modified TESLA version called TESLA++ that is more secure and efficient. In TESLA++, the sender first broadcasts the MAC, then after a short period, the sending vehicle broadcasts the whole message and the authentication key.

### Asymmetric key authentication schemes

Lin et al. (2007) integrated the identity-based signature with group signature techniques to provide security assurance with privacy preservation. Raya, Papadimitratos & Hubaux (2006) introduced a message authentication with privacy preservation protocol. In this protocol, each vehicle had a group of anonymous keys (public/private) and each key was used for a short time before being revoked. Zhu et al. (2014) proposed another efficient authentication scheme in which the whole area was divided into several regions (domains). Each region was managed by one RSU. The authors replaced the certificate revocation list (CRL) with a hash message authentication code (HMAC). This process minimized the total time required in the checking process.

### Hybrid key authentication schemes

Studer et al. (2009) proposed a VANET authentication protocol (VAST) that combined the elliptic curve digital signature algorithm (ECDSA) with TESLA++. This combination resulted in both low communication and low computation, and achieved the non-repudiation requirement. Alfadhli et al. (2020) proposed a lightweight scheme called SD2PA that overcame the problem of non-safe driving in critical areas. This scheme provides a low computational and communication overhead. Manivannan, Moni & Zeadally (2020) provided a comprehensive survey on security issues that arise on VANET, including authentication, privacy, and secure message broadcasting and shed light on the open research security issues in VANET.

Lim & Manivannan (2016) proposed an efficient, secure, authenticated message delivery protocol. This protocol is dependent on the vehicle-to-infrastructure (V2I) communication model. They used the Diffie–Hellman (DH) protocol to generate and share the symmetric keys. This protocol uses both symmetric and asymmetric key cryptography algorithms. Liu et al. (2016) enhanced the message forwarding technique proposed by Lim & Manivannan (2016) using aggregate message authentication codes instead of the onion signature. Islam, Taha & Rowayda (2017) proposed a low computation overhead protocol that used a CRT-RSA cryptography algorithm instead of a traditional RSA. This protocol generated a shared symmetric key without using the well-known Diffie–Hellman protocol. The Diffie–Hellman protocol produces a large amount of computation overhead at each node.

We propose an efficient authentication protocol for message delivery. This protocol is considered to be an enhanced version of the low computation overhead protocol proposed by Islam, Taha & Rowayda (2017). This proposed protocol utilizes a combination of symmetric and asymmetric key cryptography algorithms. Moreover, we used a symmetric key generated and exchanged without using the Diffie–Hellman protocol (Diffie & Hellman, 1976). The group key is changed from an asymmetric key to a symmetric key instead of that presented by Islam, Taha & Rowayda (2017). The proposed change significantly reduces the overall computation overhead while preserving the overall security level.

The proposed protocol utilizes CRT-RSA (Kondracki, 1997) and (CRT-RSA algorithm, 2020) instead of using RSA (Mollin, 2002) to achieve the least encryption and decryption time as CRT-RSA is much faster than both RSA and Elliptic Curve (Dindayal & Dilip, 2018).

## System assumptions and objectives

There are many assumptions required in VANET architecture as illustrated in Lim & Manivannan (2016) and Liu et al. (2016). This section summarizes the assumptions needed for testing the proposed protocol. The protocol objectives will also be illustrated in this section.

### Proposed assumptions

We divided the whole VANET area into domains (groups), and each domain has one RSU that managed group communication. We used the V2I communication model and communication was performed only through the RSU. Each vehicle, within a group, was able to send or receive messages through the RSU using a suitable routing protocol. All RSUs could communicate with each other through wired or wireless communications and could communicate indirectly through the RTA. All RSUs, RTAs, and TA were trusted to be resistant to attacks. Moreover, the security parameters of each node (Vehicle or RSU) were changed periodically.

### Objectives of the proposed protocol

Our protocol sought to achieve the following objectives:

- *Low computation and communication overhead*: The communication between vehicles and RSU remains intact for a few minutes. Hence, all required communication and computation processes should be performed quickly.
- *Message integrity*: Every message has to be delivered without any modifications and the message’s data integrity should be ensured.
- *Message authenticity*: The message source should be authenticated to prevent the impersonation attack.
- *Privacy preservation*: The real identity of vehicles should be protected during communication. However, the authorities should be able to find the real identity of any vehicle in exceptional cases, such as liability investigation.

Our notations are listed in Table 1.

**Table 1:** Notations.

Notation	Description
$R_{j}$	An RSU
$V_{i}$	A Vehicle
M	A Message
TS	A Timestamp
$Cert_{i}$	Node i’s Certificate
$ID_{p_{i}}$	The Vehicle’s real identity
$PID_{p_{i}}$	The Vehicle’s pseudo Identity
$PK_{p_{i}}$	The Public key of entity $p_{i}$
$SK_{p_{i}}$	The Private key of entity $p_{i}$
$K_{A-B}$	A Shared Symmetric key between A and B
H()	A One-Way Hash function
$dgt_{i}$	The message digital signature
$X_{pos},\;Y_{pos}$	The Location coordinates (x, y)

## The proposed protocol

**Figure 3:**
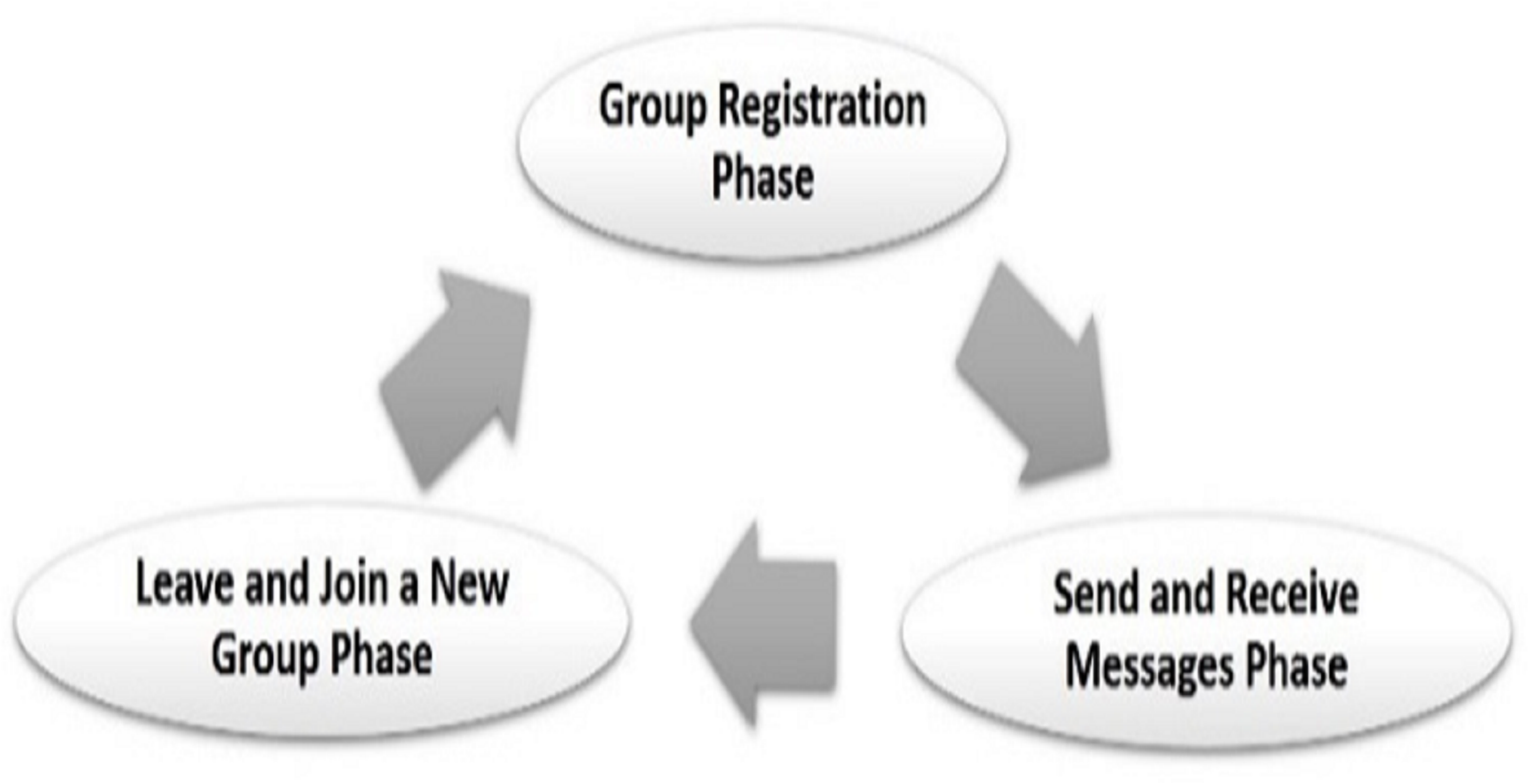
The proposed protocol phases.

The proposed protocol employs the Vehicle-to-Infrastructure (V2I) communication model. This communication model is analogous to the client-server model. Therefore, all communications have to be performed through the RSU. Moreover, there is no direct communication between vehicles (Dindayal & Dilip, 2018). However, OBUs can only communicate directly to exchange routing messages and vehicles can intercommunicate only through the RSU that controls the domain (group). The proposed protocol has three main phases: the vehicle registration phase, the send/receive message phase, and the join/leave a group phase as shown in Fig. 3. We sought to minimize the overall computation time required in each phase while preserving the security level. The computation time is critical for safety applications since the safety messages should be completely intolerant of the delay. CRT-RSA (Islam, Taha & Rowayda, 2017) was used as a public key cryptography algorithm. AES-128 was used as a private key cryptography algorithm. The CRT-RSA cryptography algorithm combined the Chinese Remainder Theorem (CRT) (Kondracki, 1997) with the traditional RSA (CRT-RSA algorithm, 2020). This combination (CRT-RSA) is more efficient in message decryption compared to traditional RSA (Islam, Taha & Rowayda, 2017).

### A group registration phase

**Figure 4:**
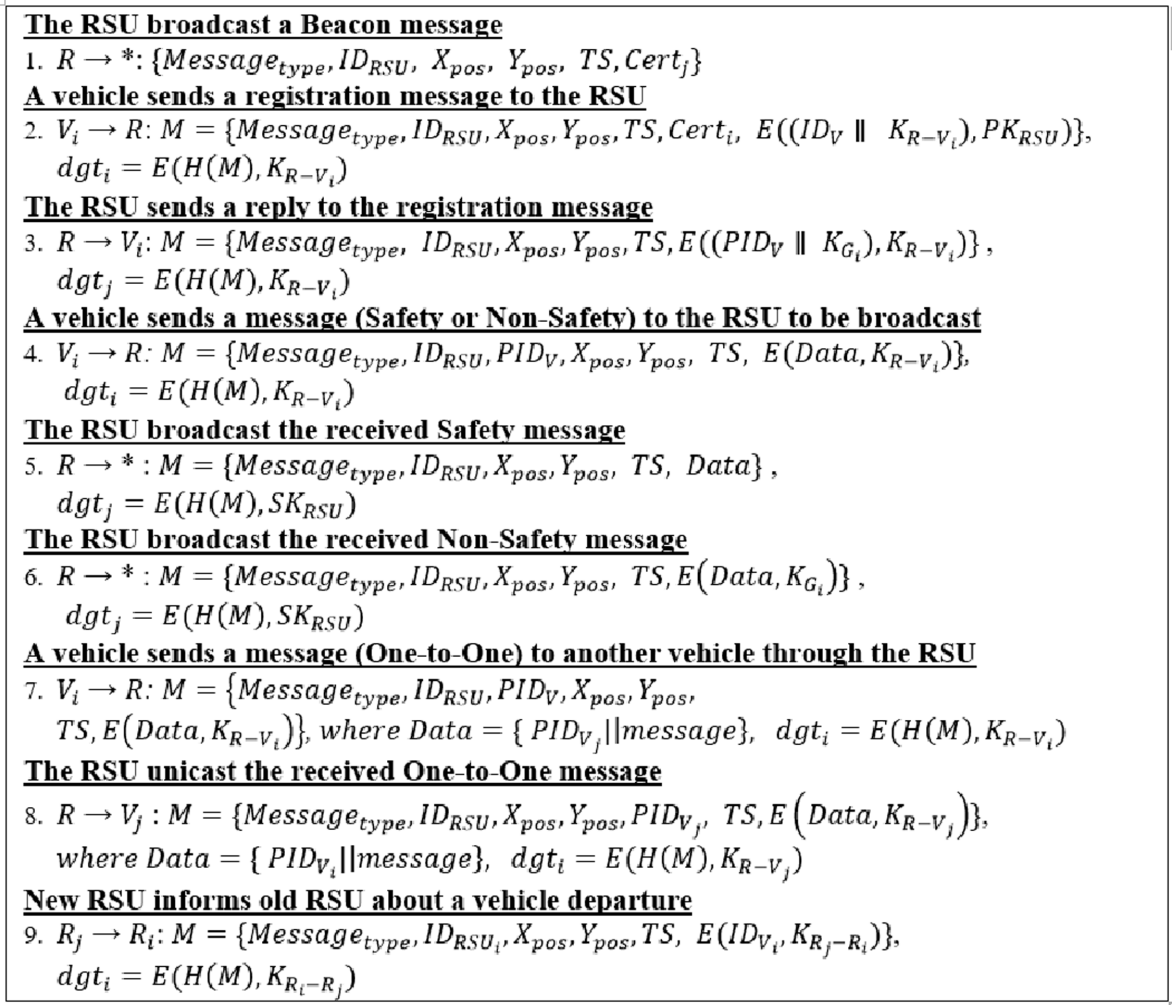
Proposed protocol.

Each RSU broadcasts a beacon message contains its coordinates (x, y), timestamp (TS), and $ID_{RSU}$, as shown in Fig. 4. This message is broadcast periodically every 100 ms (IEEE Standards Association, 2006). A vehicle produces random 128 bits when it receives this message and the bits are utilized as a shared symmetric key with the RSU. The receiving vehicle then encrypts the generated key and its real identity using the public key of the RSU. This vehicle sends the encrypted data, through a registration message, to the RSU, as shown in Fig. 4. The digital signature of the message is generated using the generated shared symmetric key ($K_{V_{i}-R_{j}}$) instead of the asymmetric key.

The group registration phase by Lim & Manivannan (2016) and Liu et al. (2016) uses the Diffie–Hellman (DH) protocol. DH is a public key cryptography algorithm that is used to produce and distribute a symmetric key between two entities (Diffie & Hellman, 1976). The key generation using DH protocol increases the computational overhead at the two entities. However, our symmetric key was generated and distributed similarly to Islam, Taha & Rowayda (2017), in which DH is not used and the vehicle generates random bits that will be used as a symmetric key. The vehicle then encrypts the generated key using the RSU’s public key before sending. Each vehicle, within the vehicle’s transmission range, can receive the encrypted message. However, only the RSU can decrypt the message because the RSU has the appropriate private key.

The RSU will extract the vehicle's real identity ($ID_{V}$) and the generated symmetric key ($K_{V_{i}-R_{j}}$) once the registration message is received. Next, the RSU will generate a random pseudo-identity ($PID_{V}$). This $PID_{V}$ is used by the message sender (vehicle) for its incoming communications instead of its real identity. The RSU will then send a reply message, which is shown in Fig. 4. A vehicle will get its new$PID_{V}$ and the shared group secret key$\;K_{G_{i}}$ when a vehicle receives a reply to its registration message. This secret key $K_{G_{i}}$ will be used in its group communications.

We changed the group key to a symmetric key rather than the asymmetric key found in the literature (Lim & Manivannan, 2016; Liu et al., 2016; Islam, Taha & Rowayda, 2017). The symmetric key algorithms provide integrity, confidentiality and authenticity. The main advantage of these algorithms is the low computation overhead and these algorithms are important in real-time applications. The symmetric key algorithms have the same security level as asymmetric key algorithms, which could be achieved with a smaller key size and lower computation overhead compared to asymmetric key algorithms (Luhach, 2016).

### Send/receive messages phase

In this protocol, the transmitted data changed according to the application type. VANET applications can be classified as safety and non-safety applications. Safety applications have strict time constraints compared to non-safety applications. In safety applications, the message should be delivered within no more than 100 ms (Yousefi, Fathy & Bastani, 2012) and the computation delay should be minimal in safety applications.

A vehicle can send a one-to-one message after completing the registration phase (Unicast) or a group message (Broadcast). A vehicle sending a group message should first send the message to the RSU that manages the group. This message will be encrypted using the shared symmetric key between the vehicle and the RSU, as shown in Fig. 4. This message can be a safety message or a non-safety message. In case of a safety message, the RSU will broadcast the message as plaintext, as shown in Fig. 4. Otherwise, the message will be encrypted using the shared group secrete key $K_{G_{i}}$ as shown in Fig. 4.

The one-to-one message is sent from a vehicle to another group member (vehicle). This message contains the data to be sent in addition to the $PID_{V}$ of the desired vehicle. The data and the destination’s $PID_{V}$ will be encrypted using the shared symmetric key ($K_{V_{i}-R_{j}}$). The message will then be sent to the RSU (Fig. 4), where it is received by the RSU and the message integrity and authenticity are verified. The RSU generates a message with the data that needs to be sent in addition to the $PID_{V}$ of the original message sender (vehicle). This message, plus the source’s $PID_{V}$, are encrypted using the shared symmetric key between the vehicle and the RSU. The RSU then sends the message as shown in Fig. 4.

### Leave/join group phase

A vehicle outside the transmission range of the RSU (that controls its group) receives a beacon message from the neighboring RSU that controls the neighbor group. The vehicle will receive two beacon messages every 100 ms from two different RSUs. The vehicle compares the value of the received signal strength indicator (RSSI) of the two received signals. This process will determine the best RSU based on the RSSI values. If the new RSSI value is higher than the old one, this means that the vehicle is moving towards the new RSU. In this case, the vehicle should send a new registration message to leave its current group and join the new group that is controlled by the new RSU, as shown in Fig. 4. When the new RSU receives the registration message, it will send an informative message to the old RSU about vehicle departure as shown in Fig. 4. The RSSI value can be calculated as in Eq. (1) (Islam, Taha & Rowayda, 2017):

(1) $$P_{r}=\frac{P_{t}*G_{t}*G_{r}*h_{t}^{2}*h_{r}^{2}}{d^{4}L}$$

Where, $P_{r}$ is the power received at distance d, $P_{t}$ is the transmitted signal power, $G_{t}$ is the transmitter gain, $G_{r}$ is the receiver gain, $h_{t}$ is the transmitter antenna height, $h_{r}$ is the receiver antenna height, and *L* is the path loss.

## Simulation results and discussion

Our experiments were performed using the NS-2.35 simulator (NS-2.35 Network Simulator, 2020) with simulation parameters illustrated in Table 2 (Islam, Taha & Rowayda, 2017). We used NS-2.35 for its robustness and maturity, which is the industry standard for simulations, experiments, and testing in other studies (Kumar et al., 2020). Although NS3 is recent compared to NS-2.35, the proposed work should be compared to previous work in (Lim & Manivannan, 2016; Islam, Taha & Rowayda, 2017) using NS-2.35. Our proposed protocol can use any existing routing protocol and the AODV protocol was selected to satisfy our requirements. We used the two-ray ground model for the propagation model for its lower packet error probability (PER) (Tripp-Barba et al., 2020). This model was suitable with the chosen AODV routing protocol (Angeles et al., 2020). All experiments were performed on a PC with Linux Ubuntu (16.04) and an Intel Core i7 processor with 4 GB RAM.

**Table 2:** Simulation parameters.

Parameters	Value
Simulation Area	1,000 m × 1,000 m
Simulation Time	400 s
Wireless Protocol	802.11p
Routing Protocol	AODV
Default Antenna Power Transmission	0.28183815
Default Antenna Height	1.5 m
Default Antenna Path Loss	1 dB
Default Antenna Gain	1 dB
Radio Propagation Model	TwoRayGround
Beacon Interval Time	100 ms
Number of Vehicles	5 Vehicles
Symmetric Key Cryptography	AES-128, SHA-256
Public Key Cryptography	DH, RSA and CRT-RSA

We evaluated the registration phase’s computation time in comparison with the protocol developed by Lim & Manivannan (2016). We reviewed the computation overhead of the send/receive group message and compared this to the protocol developed by Islam, Taha & Rowayda (2017). We also calculated the computation overhead of both the send/receive group message and the send/receive one-to-one message.

### Results of the group registration phase

We evaluated the computation overhead effect in the registration phase and compared the effects on the total time needed to complete this phase to the results from (Lim & Manivannan, 2016).

Our experiments were conducted using four different scenarios as follows:

- ***In the first scenario***, we used the protocol described in Lim & Manivannan (2016). This model used DH to generate and distribute the symmetric key during the registration process. It also used the RSA algorithm.
- ***The second scenario*** was the same as the first scenario but used the CRT-RSA instead of the RSA algorithm.
- ***In the third scenario***, the shared symmetric key was generated and distributed without using DH. This scenario used RSA as in the first scenario.
- ***In the fourth scenario***, we use the proposed protocol and the shared symmetric key was generated and distributed without using DH as in the third scenario. In addition, this scenario used CRT-RSA algorithm as in the second scenario.

**Figure 5:**
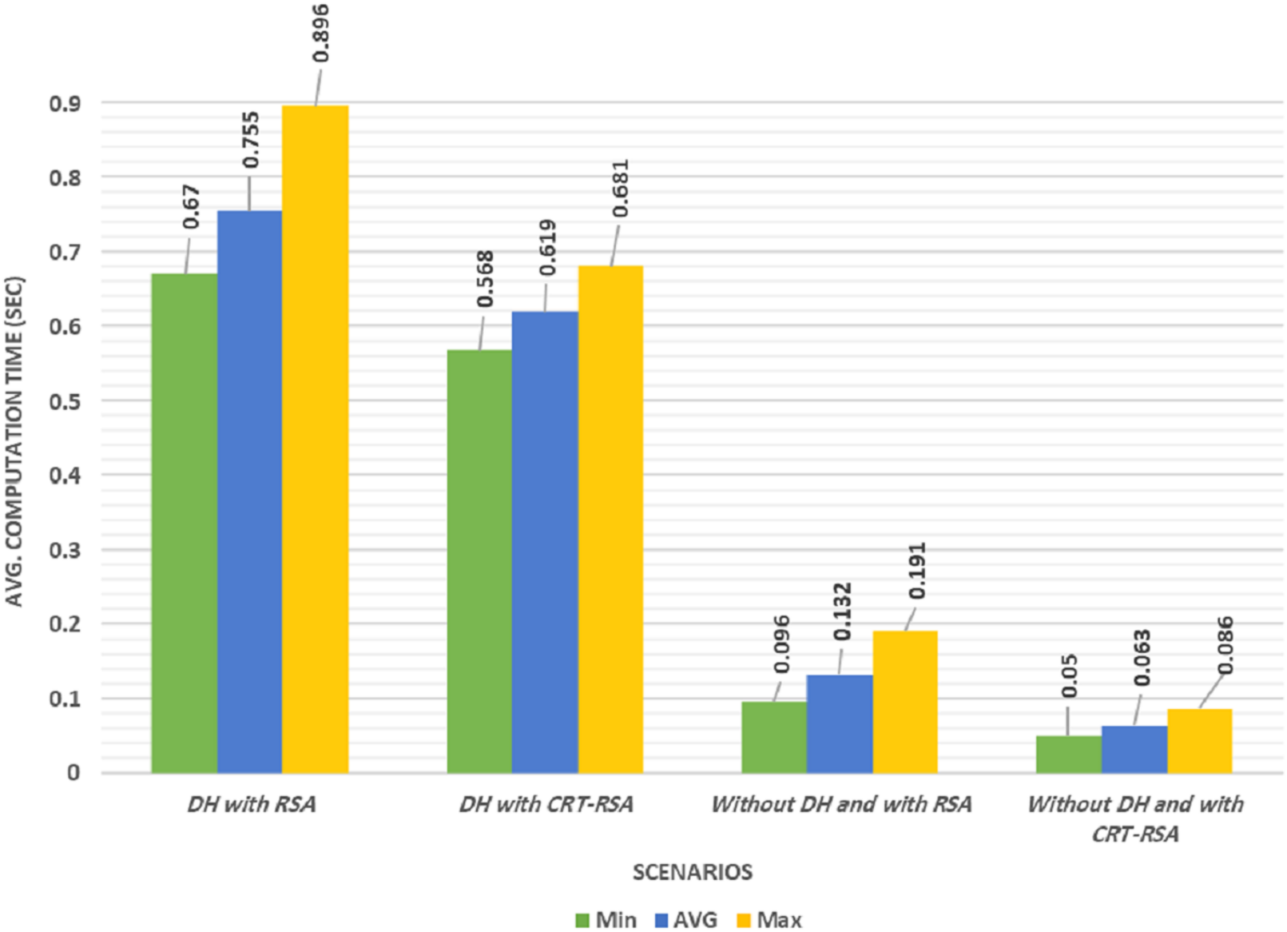
Computation time of the four different scenarios.

The computational time depended on many internal factors including memory access time, integer divisions, and multiplication time. Each scenario was simulated five times for greater accuracy, and the average, minimum, maximum, and the standard deviation (SD) values of the computational overhead were calculated from each scenario as illustrated in Table 3. Figure 5 shows the computational overhead of the registration phase in four different scenarios. The results show that the first scenario has the highest average computational delay, whereas, the fourth scenario had the lowest average computational delay. The average computational time of the second scenario was improved by 18% compared to the first scenario.

**Table 3:** Computation overhead of each scenario in seconds.

Scenario	Min	Max	SD	Avg.
using DH with RSA (Studer et al., 2009)	0.670	0.896	0.051	0.755
using DH with CRT-RSA	0.568	0.681	0.030	0.619
without using DH and with RSA	0.096	0.191	0.025	0.132
without using DH and with CRT-RSA “Proposed Work”	0.050	0.086	0.008	0.063

The third scenario’s average computational time improved by 82.5% compared to the second scenario. The fourth scenario’s average proposed computational time was enhanced by 91.7% compared to the third scenario. Our proposed protocol was the highest performing among the four scenarios studied.

### Results of send/receive group non-safety message

Our experiments were performed to evaluate the average computational time of sending and receiving a non-safety group message. We examined the average computational time required to encrypt and decrypt the non-safety message and the symmetric key results from the proposed group were compared with the works introduced by Lim & Manivannan (2016) and Islam, Taha & Rowayda (2017).

We performed our experiment using two different scenarios: in the first scenario, we used the proposed group symmetric key protocol. In this case, the RSU encrypted the group message using the shared group symmetric key $\left(K_{G_{i}}\right)$ and the RSU broadcasted the message to its group. The vehicle decrypted the message using the shared group symmetric key $\left(K_{G_{i}}\right)$ once it was received. In the second scenario, we used the group asymmetric key described by Lim & Manivannan (2016) and Islam, Taha & Rowayda (2017) where the RSU encrypted the message using the private group key $\left(SK_{G_{i}}\right)$. Then, the RSU broadcasted the message to its group members and the vehicle decrypted it using the shared public group key $\left(PK_{G_{i}}\right)$ once the message was received.

Occasionally, these two scenarios are examined in three different cases. In the first case, the message is broadcast to the current group only (zero-hop). In the second case, the message is broadcast to the current group and the adjacent group using only one-hop. In the third case, the message is broadcast to the current group and to the adjacent groups using only two-hop.

**Figure 6:**
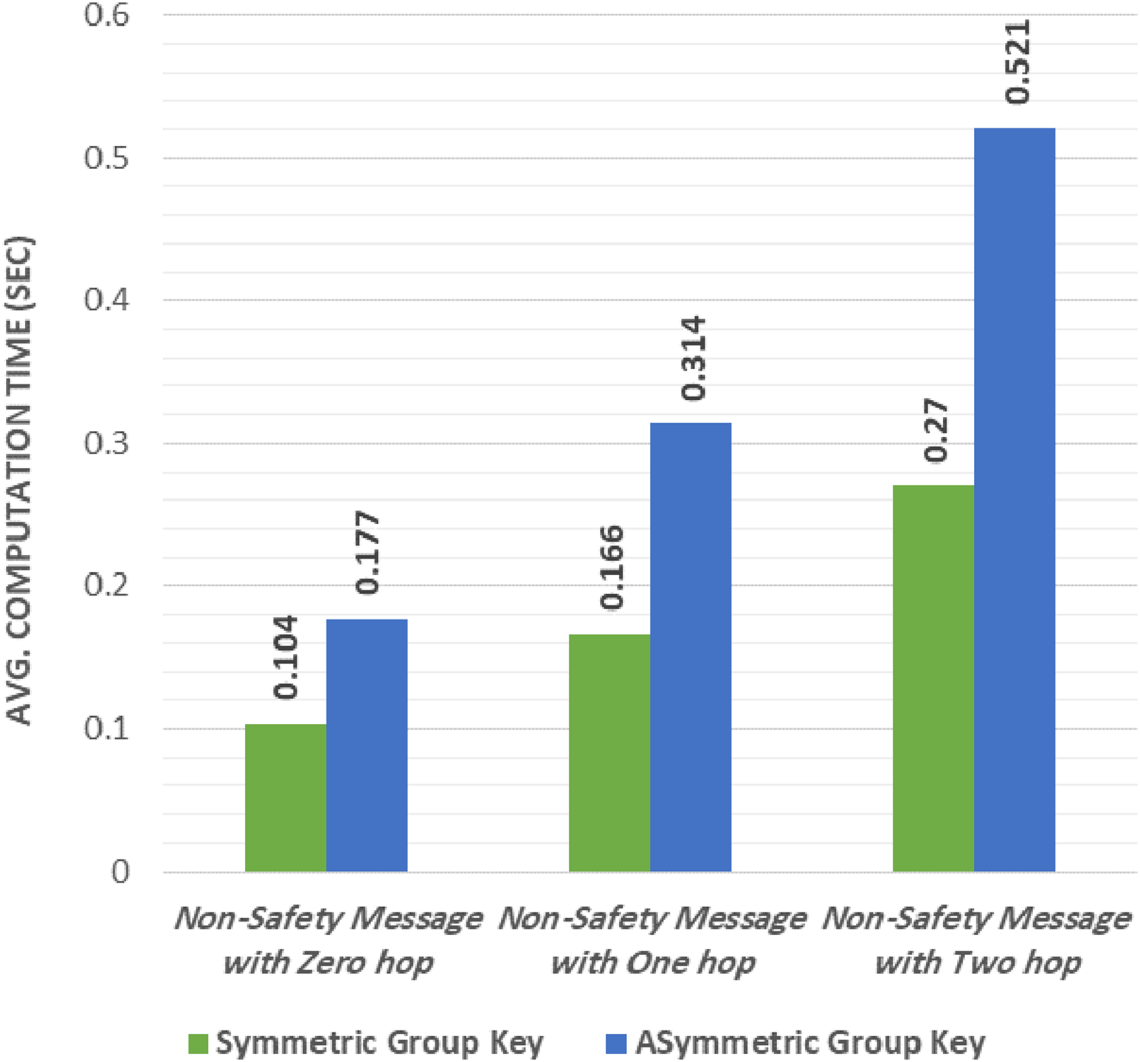
Symmetric group key vs asymmetric group key.

Table 4 and Fig. 6 show that sending and receiving a non-safety message encrypted with an asymmetric group key has the highest computational overhead. Interestingly, the computational overhead was enhanced when using the proposed group key (symmetric group key). The proposed protocol decreased the computational overhead by 41.2% in the zero-hop message. In the one-hop message, the overhead was decreased by 47.1% when using the proposed protocol. Finally, the overhead was decreased by 48.1% when using the proposed protocol with the two-hop message.

**Table 4:** Computation overhead of using symmetric group key vs asymmetric group key.

Scenario	Symmetric Group Key (Studer et al., 2009; Alfadhli et al., 2020)	Asymmetric group key “Proposed Work”
Non-Safety Message with Zero Hop	0.104	0.177
Non-Safety Message with One Hop	0.166	0.314
Non-Safety Message with Two Hop	0.270	0.521

### Results of send/receive a group safety message

**Figure 7:**
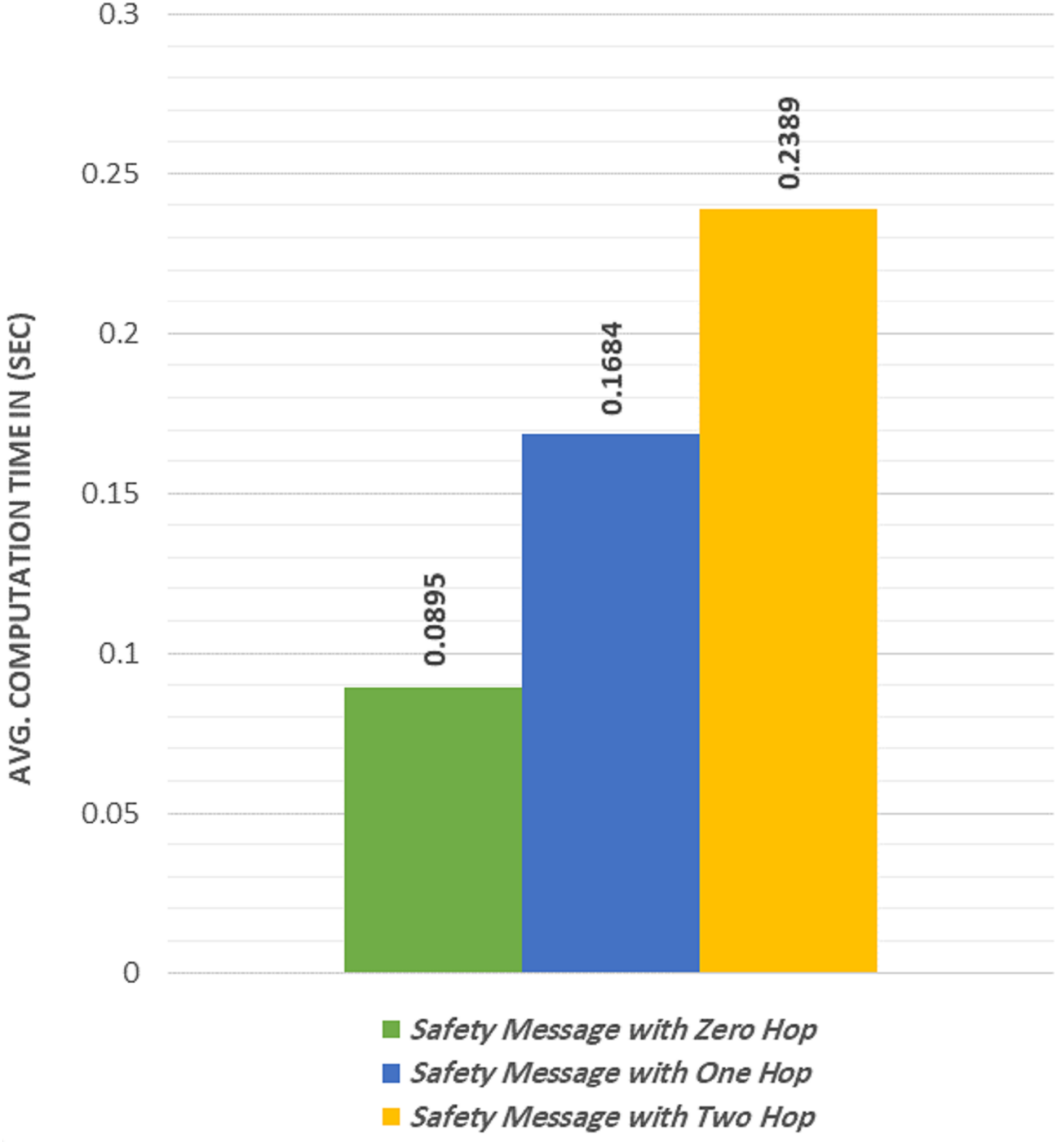
Average computation time to broadcast a safety message.

We calculated the average computational time required to send and receive a safety group message. Similar to the non-safety experiments, three different cases were used in the non-safety experiment, which were to broadcast a safety message using zero-hop, one-hop, and two-hop. Fig. 7 shows that the safety message was sent and received within a time duration less than 100 ms in the zero-hop case as constrained in (Yousefi, Fathy & Bastani, 2012). The proposed protocol satisfies the time constraint described in (Yousefi, Fathy & Bastani, 2012).

### Results of sending a one-to-one message

The proposed protocol allowed vehicles to send and receive a one-to-one message (unicast message). When a vehicle needed to unicast a message to another vehicle, that message was sent to the RSU. The RSU then checked that the destination vehicle was still a group member or if it had moved away. If the vehicle was still a member in the group, the RSU forwarded the message to the destination vehicle. If the vehicle had moved away, it forwarded the message to the RSU that managed its new group.

We calculated the average computational time required to send the message from a vehicle to another one in three different cases as follows: in the first case, the two vehicles were within the same group (zero-hop). In the second case, the destination vehicle was moved to the adjacent group (one-hop). In the third case, the destination vehicle was moved away by two-hop.

**Figure 8:**
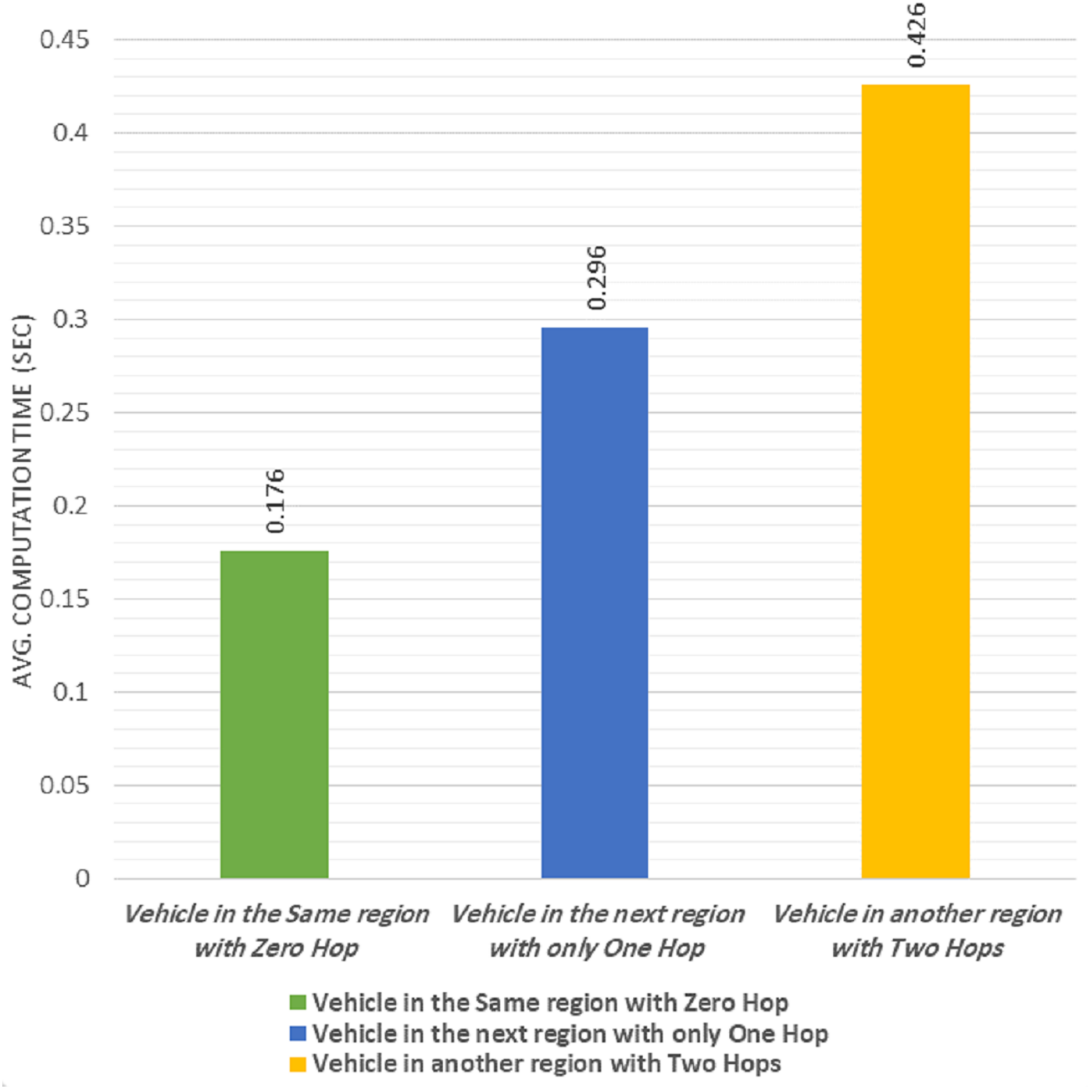
Average computation time of one-to-one message.

The experimental results are shown in Fig. 8. The average computational time required to send and receive a one-to-one message in the zero-hop, was 0.176 s, which is acceptable for non-safety applications (Hu & Laberteaux, 2006) and the proposed protocol satisfies the time recommended (Hu & Laberteaux, 2006).

## Discussion

The proposed protocol offered three different means to reduce the overall computational overhead (delay). First, a vehicle entering a new region had to register itself with the RSU managing that region and the vehicle generated random bits utilized as a shared symmetric key, sending it to the RSU. Consequently, the shared symmetric key was generated and shared without using the Diffie–Hellman cryptography algorithm. The proposed protocol minimized the overall computational time in the registration process.

Secondly, the proposed protocol utilized the CRT-RSA algorithm, which was more efficient than the traditional RSA in the decryption process. The required computational time to broadcast the non-safety messages was decreased using a shared symmetric key. It is known that the encryption and decryption processes using symmetric keys are faster than using asymmetric keys.

Lastly, vehicles sent a group message through the RSU and the verification of the message’s authenticity and integrity was performed once at the RSU. The RSU used the shared group symmetric key to encrypt the message and broadcasts the message to all other vehicles within its group. When the broadcast message was received by a vehicle, it was decrypted using the shared group symmetric key. This sequence decreases the overall computational time at the vehicle and the RSU.

## Security analysis

We examined the security analysis of the proposed work and analyzed the security requirements and resistance to attacks.

### Security and privacy features

The proposed protocol provides essential security requirements such as message authentication with privacy preservation, message confidentiality, message integrity, and non-repudiation.

#### Message authentication with privacy preservation

In the proposed protocol, a vehicle received a new random pseudo-identity ($PID_{V}$) every time it registered itself at the RSU that managed its region. The new identity was utilized instead of its real identity ($ID_{V}$) in the group communication. This process preserved the real identity of the vehicle in the group. Each RSU created a table that held information about each vehicle in its group. This information included each vehicle's certificates, the group symmetric key, the real identity, and each registered vehicle's shared symmetric key. This information was used to verify the message source authenticity. Message authentication was performed and privacy was preserved in the proposed protocol.

#### Message confidentiality

In the proposed protocol, we encrypted the content using a suitable shared symmetric key. In the case of sending the message to a specific RSU, only that RSU had the correct key to decrypt the message. In case of broadcasting a non-safety message to all vehicles in the group, only registered vehicles owning the group symmetric key were able to decrypt the received message, which ensured message confidentiality. In case of broadcasting a safety message, each vehicle (registered or not) in the group is able to receive the plaintext message, allowing them to use the information contained in the message.

#### Message integrity and non-repudiation

Each sent message in the proposed protocol had a digital signature used to verify the message integrity and authenticity. This digital signature was generated by computing the hash value of the whole message. Then, the message was encrypted using the shared symmetric key. The hash value had two main benefits: first, it could be used to check message integrity, and second, it reduced the time required to generate the message signature due to its small size. The non-repudiation issue could be handled by encrypting the message signature using the shared secret symmetric key. Checking the message integrity resisted an attack by message fabrication, in which the attacker tries to alter the message contents.

### Attack resistance

VANET and WSN both receive the same attack methods, including Sybil Attack, Message Fabrication and Modification Attack, and Eavesdropping Attack (Quyoom, Mir & Sarwar, 2020; Tchórzewski & Jakóbik, 2019). Our proposed work can resist many of these attacks. The main difference between WSN and VANET in security attacks is the battery drainage attacks which are a critical issue in WSN. An example of such attacks is the Denial-of-Sleep Attack.

#### Fake beacon attack

The proposed protocol resisted the fake beacon attack by preventing malicious nodes from appearing as real RSUs. In the proposed protocol, each vehicle was preloaded with useful information about each RSU, including the locations of each RSU, $Cert_{RSU\;},\;ID_{RSU}$ and $PK_{RSU}$. The communication between the vehicle and the RSU in the registration process was encrypted with $PK_{RSU}$ and the malicious node cannot obtain useful information without using the correct $SK_{RSU}$. Consequently, the proposed protocol prevented malicious nodes from sending fake beacon messages.

#### Sybil attack

This attack occurs when a malicious vehicle poses as multiple vehicles and sends false information. The proposed protocol can resist this attack. Each vehicle must register itself using its certificate ($Cert_{V}$) at the RSU that controls the group. Then, the RSU checks the validity of the certificate. A valid certificate allows the RSU to generate a pseudo-identity ($PID_{V}$) for this vehicle and will save this information in the vehicle list. An invalid certificate caused the RSU to ignore this registration request so that a malicious vehicle cannot register with a fabricated certificate. A unique secret symmetric key is shared between the RSU and each vehicle in the group to prevent a malicious vehicle from sending a fake message on behalf of a registered legal vehicle.

#### Message fabrication and modification attacks

These types of attacks aim to insert fake messages by unauthorized nodes. In the proposed protocol, each message sent by a legal vehicle was hashed and encrypted using the shared symmetric key and the vehicle sent the message to the RSU. The RSU then checked the integrity of the message to ensure that the message was not tampered with by any other vehicle during transmission. Our proposed protocol resists these types of attacks.

#### ID disclosure attack

This type of attack aims to obtain the real identity of a vehicle by tracking its current location. In the proposed protocol, each vehicle received a $PID_{V}$ during its registration process. This $PID_{V}$ was used instead of its real identity. All broadcast messages were sent by the RSU itself on behalf of the originating sender. Every time a vehicle entered a new region, it received a new $PID_{V}$ that was completely different from the previous one. This process prevented location tracking attacks.

#### Eavesdropping attack

Eavesdropping is a passive attack that aims to get sensitive information by listening to the traffic. It is also known as a snooping or sniffing attack. In our proposed protocol, all sensitive data within a unicasted messages were encrypted using the shared symmetric key ($K_{V_{i}-R_{j}}$). The broadcast messages were encrypted using the group key ($K_{G_{i}}$) and message confidentiality was ensured in the proposed protocol.

#### Collision attack

The collision attack threatens the hash function by attempting to find two inputs that produce the same hash value. In the proposed protocol, we used SHA-256 as the hash function which achieved a good security level with a low computational overhead. SHA-256 had a 128-bits security level for collision and 256-bits for pre-image (Tchórzewski & Jakóbik, 2019), indicating that the number of $2^{128}$ trials were required to break SHA-256 with a collision attack. These trials posed a large challenge to processing units. Additionally, the security parameters of each node in the proposed protocol periodically change so that even if an attacker were able to crack the hash function, the proposed protocol is still secure due to frequent changes in the security parameters. Consequently, this attack is unrealistic in case of using the proposed protocol.

#### Replay attack

In this attack, an attacker delays a valid message that has been sent early and a prior valid message can be repeated in order to make some disturbances in the traffic. For example, the attacker can resend an old alarm message to other vehicles. In this case, the vehicles will receive false information and the vehicles may make wrong decisions. The proposed protocol can handle this issue by attaching a timestamp to each sent message to ensure the freshness of the messages to prevent this type of attack by validating the attached timestamp.

#### Denial-of-sleep attack

This attack aims to reduce the battery life of each node. This attack primarily targets WSN and reduces the network life span (Fotohi & Bari, 2020). Any vehicle in VANET has enough energy to sustain computation and communications; the energy consumption of VANET is not a concern when compared with WSN. As a consequence, this attack is not significant in VANET.

## Conclusion

We proposed a novel message delivery and authenticity protocol that preserved privacy. Our protocol used an efficient combination of symmetric and asymmetric cryptography algorithms. For the asymmetric key, we used an efficient version of the RSA protocol called CRT-RSA. For the symmetric key, AES-128 was used. The proposed protocol efficiently reduced the time required for the decryption process and the symmetric key was generated and distributed without using the DH protocol that results in high computational time overhead. The shared group asymmetric key was changed to be symmetric in our proposed protocol, to achieve the required security level with a minimal computational time.

Results obtained from the simulations show that the total computational time at each node was significantly reduced using the proposed protocol. The proposed protocol reduces the average computational time by 91.7% of the computational overhead in the registration phase compared to other work in the literature. For sending and receiving a non-safety group message, the total time was reduced by 41.2% compared to other protocols in the literature, while satisfying the time constraint for sending and receiving a safety message.

The proposed protocol meets all essential security requirements, including message integration, authentication with privacy preservation, confidentiality, and non-repudiation. Additionally, the proposed protocol is resistant to the fake beacon, Sybil, message fabrication and modification, ID disclosure, Eavesdropping, Collision, and replay attacks.

## Supplemental Information

10.7717/peerj-cs.519/supp-1 Supplemental Information 1 Source Code. Sorce Code (.h and .c files)

## Additional Information and Declarations

### Data Availability

The following information was supplied regarding data availability: The source code is available as a Supplemental File.
